# Replicability of neural responses to speech accent is driven by study design and analytical parameters

**DOI:** 10.1038/s41598-021-82782-4

**Published:** 2021-02-26

**Authors:** C. Benjamin Strauber, Lestat R. Ali, Takako Fujioka, Candace Thille, Bruce D. McCandliss

**Affiliations:** 1 Stanford University, Stanford, USA; 2 Harvard Medical School, Boston, USA

**Keywords:** Language, Perception

## Abstract

Recent studies have reported evidence that listeners' brains process meaning differently in speech with an in-group as compared to an out-group accent. However, among studies that have used electroencephalography (EEG) to examine neural correlates of semantic processing of speech in different accents, the details of findings are often in conflict, potentially reflecting critical variations in experimental design and/or data analysis parameters. To determine which of these factors might be driving inconsistencies in results across studies, we systematically investigate how analysis parameter sets from several of these studies impact results obtained from our own EEG data set. Data were collected from forty-nine monolingual North American English listeners in an event-related potential (ERP) paradigm as they listened to semantically congruent and incongruent sentences spoken in an American accent and an Indian accent. Several key effects of in-group as compared to out-group accent were robust across the range of parameters found in the literature, including more negative scalp-wide responses to incongruence in the N400 range, more positive posterior responses to congruence in the N400 range, and more positive posterior responses to incongruence in the P600 range. These findings, however, are not fully consistent with the reported observations of the studies whose parameters we used, indicating variation in experimental design may be at play. Other reported effects only emerged under a subset of the analytical parameters tested, suggesting that analytical parameters also drive differences. We hope this spurs discussion of analytical parameters and investigation of the contributions of individual study design variables in this growing field.

## Introduction

In a rapidly globalizing world, individuals increasingly interact with people who differ from them by linguistic, cultural, and national background. One feature that prominently varies across individuals from these different backgrounds when they communicate in the same language is accent, a feature of speech that can drive both miscommunication^1,2^ and social judgment^3–5^. While decades of research have examined how speech is processed by the brain^6,7^, relatively little is known about how accent impacts this processing.

Spurred by the increasing societal relevance of this topic, several recent studies have attempted to identify differences and similarities that may exist in neural processing of speech in an accent that either matches or does not match the accent of the listener^8–13^, referred to here respectively as in-group and out-group accents. As a note before proceeding, studies in this literature often draw a distinction between so-called “native-accented” and “foreign-accented” speech. However, in our study’s case in particular and in many other cases, a speaker can be from a country foreign to the listener but also speak the language of focus at a native or native-like level. We have thus decided not to use the native/foreign distinction here, instead following the convention of Jiang et al.^14^ by referring to the accents of focus as in-group and out-group accents. This places focus on the relationship of the speaker and the speaker’s accent to the listener while avoiding making judgments about the nativeness of the speech.

A number of studies on the neural processing of speech accent have focused specifically on electrophysiological correlates of semantic mismatch as indexed by N400, an event-related potential (ERP) component often examined in studies of language^15^. N400 is a negative-going wave, appearing approximately 400 ms after a target stimulus, that is associated with the buildup of semantic expectation and its violation. It has classically been observed to be most negative to violation of semantic expectation, as in sentences like “I take my coffee with cream and dog”^16^. Studies examining neural correlates of semantic processing in different accents have reported mixed results, with some finding a larger N400 to semantic congruence or semantic incongruence for out-group speech as compared to in-group speech, others finding a smaller N400, and still others finding no difference.

### Congruence in the N400 range

Goslin et al.^8^ found that N400 to semantically congruent sentences was significantly more negative for speech in two different accents of the listener's country as compared to speech in the accent of a different country. By contrast, Romero-Rivas et al.^10^ found that in their study’s first experimental block, which only included semantically congruent sentences, out-group speech elicited a more negative N400 than in-group speech, but this difference disappeared by the second experimental block. They interpreted this as indicating that lexical-semantic processing of out-group speech improved over time with exposure.

### Incongruence in the N400 range

Hanulíková et al.^9^ found that semantically incongruent sentences evoked a larger negative amplitude in the scalp’s posterior region than semantically congruent sentences in both in-group and out-group speech. This negativity was larger in the posterior region than the anterior region for in-group speech but not for out-group speech. Consistent with this, they found that the difference in amplitude evoked in the anterior region by semantically incongruent as compared to semantically congruent sentences trended toward significance in in-group speech but was reliably significant in out-group speech. They took these results together to suggest there may be a broader topographical distribution for the N400 effect in out-group speech as compared to in-group speech. Romero-Rivas et al.^10^ found that for in-group speech, semantically incongruent sentences elicited a more negative N400 than semantically congruent sentences only in the posterior region. By contrast, for out-group speech, semantically incongruent sentences elicited a more negative amplitude than congruent sentences in this time range in posterior, central, and frontal regions. This is consistent with the broader topographical distribution for semantic incongruence in out-group speech observed by Hanulíková et al.^9^. The N400 amplitude elicited by semantically incongruent sentences in out-group speech was more negative than those in in-group speech overall and in each of the three regions separately. Contrasting with these previous studies, Grey and van Hell^12^ found that semantically incongruent sentences elicited a more negative signal in the N400 time range than congruent sentences in in-group speech but not in out-group speech.

### Late responses to semantic incongruence

Several studies note responses to semantic incongruence later than the classical N400 window, though their results diverge on what they observe in this late window. As noted by Romero-Rivas et al.^10^, a visual inspection of the results presented by Hanulíková et al.^9^ reveals a positive deflection in a post-N400 time range (600–900 ms) that they refer to as P600 in posterior electrodes in semantically incongruent as compared to congruent sentences in in-group speech but not out-group speech, though Hanulíková et al. did not analyze or discuss this effect. Romero-Rivas et al.^10^ note that a positive-going wave in this P600 range could reflect meaning reanalysis, mentioning that a lack of a wave there for out-group but not in-group speech may suggest listeners are more tolerant of semantic violations in out-group speech, in which they do not attempt to find alternative meanings.

Looking at this time range in their own data set, Romero-Rivas et al.^10^ found that for in-group speech, semantically incongruent sentences elicited a more positive response than semantically congruent sentences, but no such congruity effect occurred for out-group speech. They also found that for incongruent sentences, in-group speech elicited a more positive response than out-group speech, but no such accent effect occurred for congruent sentences. Additionally, for sentences of both congruity types combined, they found that the P600 amplitude was significantly more positive for in-group as compared to out-group speech in the posterior region but not in the other regions, and that for both accents, the positivity was greatest in the posterior region. They suggest that reduced positivity to incongruence in out-group speech indicated a lack of an attempt to find alternative meanings for the semantic anomaly, or that listeners lacked the processing resources to find an alternative meaning.

In contrast with Romero-Rivas et al.^10^, Grey and van Hell^12^ found that semantically incongruent sentences evoked a late negativity in the 500–900 ms range for both accents. They discuss that this could either reflect a delayed N400 effect or that it could reflect conceptual rather than merely lexical processing.

In Holt et al.^13^, who performed a cluster-based analysis instead of using a priori analysis parameters, clusters reflecting a significant negativity for incongruent as compared to congruent sentences were identified between 200 and 900 ms for in-group speech but between 560 and 660 ms in out-group speech. While care must be taken in interpreting cluster-based analyses as they do not establish the temporospatial boundaries of the detected effect^17^, this observation is consistent with a late negativity to semantic incongruence in out-group speech.

### Potential sources of differences across studies

It is clear from the above discussion that observations of electrophysiological responses to semantic congruence and incongruence in in-group and out-group speech vary greatly across studies. Grey and van Hell^12^ ascribe their results’ divergence from previous studies to differences in the participant populations, as their study focused on monolinguals with limited foreign accent experience, while Hanulíková et al.^9^ and Romero-Rivas et al.^10^ employed bilinguals listening in their native language. Holt et al.^13^ suggest that discrepancies in outcomes among studies including their own could result from differences in participant population, which in their study was drawn from a cosmopolitan city with presumably high exposure to different accents, and from other studies’ use of time ranges and regions that were either pre-determined or selected from visual inspection of results. However, they do not subject their data to any of the analyses used by previous studies, so it is unclear whether they would have gotten the same results had they done so.

To this point, one major potential source of disagreement in results among these studies is differences among analytical parameters, which vary widely across the studies discussed above. For example, to look at N400, Romero-Rivas et al.^10^ use a time window from 250 to 600 ms, while Hanulíková et al.^9^ and Grey and van Hell^12^ use a time window from 300 to 500 ms. All of these studies also vary by the regions they define and the electrodes they put in each region. (Parameters used in each study, including time ranges and region definitions, can be found in Table 1). Meanwhile, the cluster-based analysis used by Holt et al.^13^ identifies that differences in signal evoked by semantically incongruent as compared to semantically congruent sentences appear reliably in out-group speech only between 560 and 660 ms. If it is the case that out-group speech produces a late N400 or some other semantically modulated difference in signal around this time frame, the fact that some studies overlap with it and others do not could lead to significantly different results among studies, even with similar data sets. For example, it is plausible that a critical component of the response to congruity manipulations in out-group speech could fall between 500 and 600 ms, as consistent with the findings of Holt et al.^13^. If this is the case, it could potentially explain the conflict between the observation by Grey and van Hell^12^ of no differential response to congruence and incongruence in the N400 window in out-group speech, and the observation by Romero-Rivas et al.^10^ of both a differential response in out-group speech and a greater N400 response to incongruent sentences in out-group speech than in-group speech, as the latter study included this 500–600 ms range and the former did not.

**Table 1:** Analytical parameters used in each ERP study of semantic congruence in accented speech.

	Regions used	Time ranges analyzed
Hanulíková (2012)^9^	1. Frontal (Fp1/2, F3/4, F7/8, FC1/2, FC5/6, F9/10, FT9/10, Fz), 2. Posterior (CP5/6, CP1/2, P7/8, P3/4, PO9/10, O1/2, Pz, Oz)	N400 (300–500 ms), P600 (800–1200 ms)
Goslin (2012)^8^	1. Midline (Fz, FCz, Cz, CPz, Pz), 2. Outer circle (FP1, FP2, F7, F8, T7, T8, P7, P8, O1, O2) 3. Inner circle (F3, F4, FC3, FC4, C3, C4, CP3, CP4, P3, P4)	PMN (200–350 ms), N400 (350–600 ms)
Romero-Rivas (2015)^10^	1. Frontal (F3, Fz, F4, FC5, FC3, FC1, FC2, FC4, FC6) 2. Central (C3, C1, Cz, C2, C4, CP3, CP1, CP2, CP4) 3. Posterior (P5, P3, P1, Pz, P2, P4, P6, PO3, PO4)	P200 (150–250 ms), N400 (250–600 ms), P600 (600–900 ms)
Romero-Rivas (2016)^11^	1. Frontal (F3, Fz, F4, FC1, FC2, FC5, FC6) 2. Central (C3, Cz, C4, CP1, CP2, CP5, CP6) 3. Posterior (P3, Pz, P4, PO1, PO2, O1, O2)	N400 (250–450 ms), P600 (500–900 ms)
Grey (2017)^12^	1. Frontal (Fz, F3, F4, FC5, FC6) 2. Central (Cz, C3, C4, CP1, CP2) 3. Posterior (Pz, P3, P4, P7, P8)	N400 (300–500 ms), P600 (500–900 ms)
Holt (2018)^13^	No a priori regions used (clusters)	No a priori time ranges used (clusters)

Another potential source of inconsistency in the results of these studies is variation in a collection of factors relevant to study design. The factors potentially driving this variation are many, as studies so far in this literature differ on a number of features that could plausibly cause major differences in observed outcome. These include:

- The homogeneity of the community in which listeners live and their presumed amount of experience with out-group accents;
- The number of participants;
- The number of sentences used, which could influence habituation both to the out-group accent and to semantic violations;
- The number of speakers;
- The native language of the speaker(s), including the phonological properties and social status of speaker accent;
- The perceived strength and intelligibility of the out-group accent;
- The native language of participants;
- The number of languages spoken by participants;
- The cloze probability of sentences, shown by Romero-Rivas et al.^11^ to differentially influence responses to in-group and out-group speech;
- The type of incongruence modulated, whether only semantic or also syntactic;
- Other sentence properties like length;
- Critical word position in sentence;
- Task during the EEG recording.

One way of gaining deeper insight into the sources of variation in results in these studies would be to examine the same data set using the variety of analytical approaches that have been used in the studies mentioned. In doing this, we would start with the assumption that differences across studies are driven by differences in analytical parameters, experimental design variables, or both, but not by any other factors. Though we would only be able to derive firm conclusions about our own study in relation to other studies, our result could provide motivation to examine the role of either or both of these potential causes in future work. If analytical parameters have played the defining role in driving differences in outcomes across studies, we would expect that using each different parameter set would lead to a qualitatively different set of results mirroring the study from which the parameter set is derived. If not, we might expect to see similar outcomes regardless of analytical technique; this would suggest that differences between our study and other studies result from experimental design variables. Alternatively, we might see varied outcomes across analytical parameters that do not match the results of the studies whose analytical parameters are used to derive them. This would suggest both that analytical parameters can drive important differences in observed results and also that differences in outcome between this study and other studies are the result of experimental design factors. We believe such an exploration of a data set is valuable at this point in the development of the literature on electrophysiological correlates of accent processing, as it is as yet unclear to what extent accent modulates the temporal and spatial nature of electrophysiological processing of semantic information.

We generated a data set suited to this exploration by recording the neural responses of a group of monolingual American English listeners to semantic congruence and incongruence in American-accented and Indian-accented speech. We report here how outcomes resulting from analyzing these data through different studies’ parameter sets compare to each other and to the results of the original studies.

## Results

**Figure 1:**
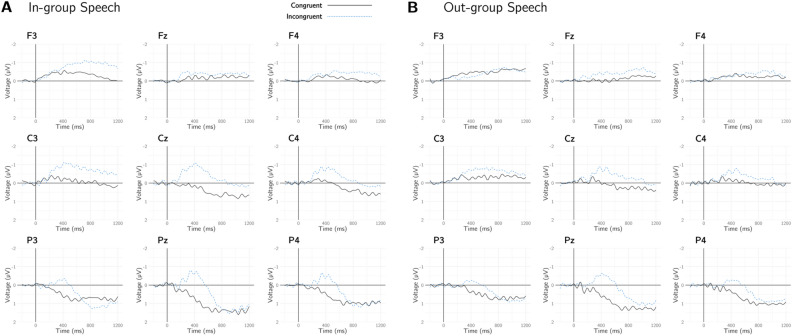
Average responses to semantic congruence and incongruence in in-group and out-group speech for three frontal, three central, and three posterior electrode sites.

To provide a general picture of the data agnostic of parameter set, we show waveforms from between 200 before and 1200 ms after the critical (congruent or incongruent) word averaged across participants in nine electrode sites. Results from this analysis are shown in Fig. 1.

Next, we analyzed our data using analytical parameters from five studies examining responses to semantic violations in in-group and out-group speech: Hanulíková et al.^9^, Romero-Rivas et al.^10^, Romero-Rivas et al.^11^, Grey and van Hell^12^, and Holt et al.^13^. We attempted to replicate the set of analytical parameters (time ranges and region maps detailed in Table 1) from each study as closely as possible. In four cases, our EEG setup was missing electrodes used in an analysis. In one case (F9/F10 in Hanulíková et al.^9^), the analysis included other electrodes that in our system were fully lateral and adjacent to where the missing electrodes would have been, meaning they should in theory have captured much of the signal that would have been captured by the missing electrodes. In the three other cases (PO9/PO10 and FT9/FT10 in Hanulíková et al.^9^, and PO1/PO2 in Romero-Rivas et al.^11^), we substituted electrodes for the nearest possible electrodes available in our setup (PO7/PO8 for PO9/PO10, FT7/FT8 for FT9/FT10, and PO3/PO4 for PO1/PO2).

We discuss here the results of ANOVAs and follow-up analyses conducted on our study’s data set in comparison with each other and in comparison with the ANOVAs and follow-up analyses reported by the other studies of focus here. We focus on two main a priori time windows defined in these studies: the N400 window and a post-N400 window. Even though not all studies of focus here found a positivity in response to semantic violations in this post-N400 window, we refer to it throughout this paper as the P600 window, following the convention of Romero-Rivas et al.^10^. All ANOVAs conducted on our data set used the factors Accent, Congruence, and Region. A separate ANOVA was conducted on our data set for each set of analytical parameters and for each time window. Main effects and interactions from all ANOVAs conducted on our data set, along with analogous ANOVA results provided by the other studies of focus, can be found in bar charts in the N400 and P600 sections as well as Supplementary Tables 1 and 2. Averages by condition can be found in Supplementary Tables 3 and 4. We present separate results using a priori parameters for the N400 time range and for the P600 time range, as well as results from a cluster-based permutation test. Because this literature has been most concerned with examining similarities and differences in ERP responses to different accents, we focus primarily on discussing main effects and interactions involving accent. As Holt et al.^13^ used a cluster-based permutation test rather than a priori analytical parameters, our replication of their analysis is presented separately from the ANOVA results, near the end of the Results section.

There are a few points regarding comparisons with existing studies that deserve particular mention. First, Hanulíková et al.^9^ conducted separate ANOVAs for each accent, so it is unfortunately not possible to make a direct comparison between our and their main effect and interaction results for either time window. Second, Romero-Rivas et al.^11^ compared best-completion words with semantically unrelated words, the latter of which may be difficult to compare with semantic violations. Third, because Romero-Rivas et al.^11^ used a between-subjects design, they conducted an ANOVA for Accent × Region on the difference wave between best-completion and unrelated words. As this is conceptually analogous to interacting these factors with Congruence, in bar charts in the N400 and P600 sections we represent the ANOVA results presented in their paper (Accent, Region, and Accent × Region) as interacted with Congruence, with the caveat that the designs of all other studies presented here, including ours, are within-subjects. Though it is thus difficult to compare some of the results from these studies to ours, we nonetheless found it informative to include them here, both to provide a healthy variety of parameter sets allowing us to examine how parameters can drive variation in reported results, and also to be able to compare their results to ours where possible.

Because we mention each study’s parameters and results frequently throughout the remainder of the Results section, to streamline discussion of each study’s parameters and results in this section, we use the following abbreviations derived from the initial of the first author’s last name and the study’s year of publication: Hanulíková et al.^9^ is H12, Romero-Rivas et al.^10^ is RR15, Romero-Rivas et al.^11^ is RR16, Grey and van Hell^12^ is G17, and Holt et al.^13^ is H18.

### A priori N400 range

**Figure 2:**
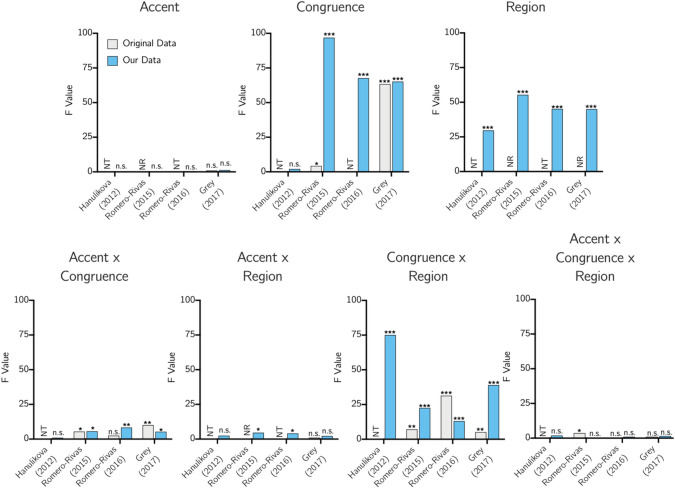
A graphical representation of F values for each main effect and interaction in the N400 time range found using each parameter set, either as reported in the original study (“original data”) or using our data (“our data”). Stars indicate significance (*p < 0.05, **p < 0.01, ***p < 0.001), “n.s.” indicates not significant at *p* < 0.05, “NR” indicates the main effect or interaction was tested in the original study but a significant result or F value was not reported, and “NT” indicates testing of the main effect or interaction was not conducted in the original study in a way comparable with our approach.

We show F values for the ANOVA of Accent × Congruence x Region in the N400 time window presented in each of the studies when available, as well as the F values calculated using each study’s parameters with our data set, in Fig. 2.

As in all studies cited, we found no main effect of Accent using any of the four sets of a priori analytical parameters.

Consistent with RR15, RR16, and G17, we found a main effect of Congruence when using the analytical parameters of each of those studies, with semantically incongruent sentences generating more negative amplitudes than semantically congruent sentences. The Congruence effect reported by G17 (F = 63.41) was much larger than the effect reported by RR15 (F = 4.25); the effect found in our study (with F values across parameter sets between 65 and 100) is closer to that reported by G17. Interestingly, we did not find a significant main effect of Congruence when using the H12 parameters. A main effect of Congruence was not discussed in their paper, as the authors do not report main effects. Closer examination revealed this discrepancy with the other parameter sets likely occurs in part because H12’s use of two regions, one that exclusively comprises frontal electrodes and another that comprises a mix of central and posterior electrodes, weights the analysis more heavily than the other studies toward the frontal region, where the congruence effect is in the opposite direction of the central and posterior regions. Their parameter set also misses many of the central electrodes, where in our data set the congruence effect appears to be the strongest.

Consistent with RR15, RR16, and G17, we found a significant interaction of Accent and Congruence using the parameters of these studies. Follow-up analyses revealed that congruent sentences spoken by the Indian-accented speaker elicited more negative amplitudes than congruent sentences spoken by the American-accented speaker, though this was only significant at *p* < 0.05 for the RR15 parameter set (*p* = 0.035) and not the RR16 (*p* = 0.08) or G17 (*p* = 0.33) parameter sets. Interestingly, our results match those of RR15 on this point when using their parameter set but not when using the other parameter sets. Incongruent sentences spoken by the American speaker elicited significantly more negative amplitudes than those generated by the Indian speaker, a difference significant at *p* < 0.05 for each of these three parameter sets. This is consistent with G17 but not RR15, who found that semantically incongruent sentences elicited a more negative amplitude when spoken in an out-group accent than an in-group accent. Incongruent sentences were significantly more negative than congruent sentences in both accents for the data analyzed with these three parameter sets. This latter finding is consistent with H12, RR15, and RR16 but not with G17, who found that only in-group-accented speech elicited a greater negativity to semantic incongruence as compared to congruence in the N400 time window. We did not find a significant interaction of Accent and Congruence using the parameters of H12. They report results of two ANOVAs conducted separately for anterior and posterior regions using the factors Accent and Congruence, with neither finding a significant interaction (F < 1). However, while our lack of a significant interaction arises because there is no congruence effect for either accent using their parameters, their lack of a significant interaction arises because there is a congruence effect for both accents.

There was a significant interaction between Accent and Region for the RR15 and RR16 parameter sets, with a trend toward a significant interaction for the other two parameter sets (*p* < 0.15). This is inconsistent with RR15, RR16, and G17, none of which report a significant interaction. Amplitudes in the frontal region were more negative for the American accent as compared to the Indian accent for all parameter sets, with this difference significant at *p* < 0.05 for the RR15, RR16, and G17 parameter sets and trending toward significance for the H12 parameter set (*p* < 0.11). No significant differences were found between accents in the central region. Amplitudes in the posterior region were more negative for the Indian accent as compared to the American accent, with this difference significant at *p* < 0.05 for the RR15 and RR16 parameter sets and trending toward significance (*p* < 0.10) for the H12 and G17 sets.

**Figure 3:**
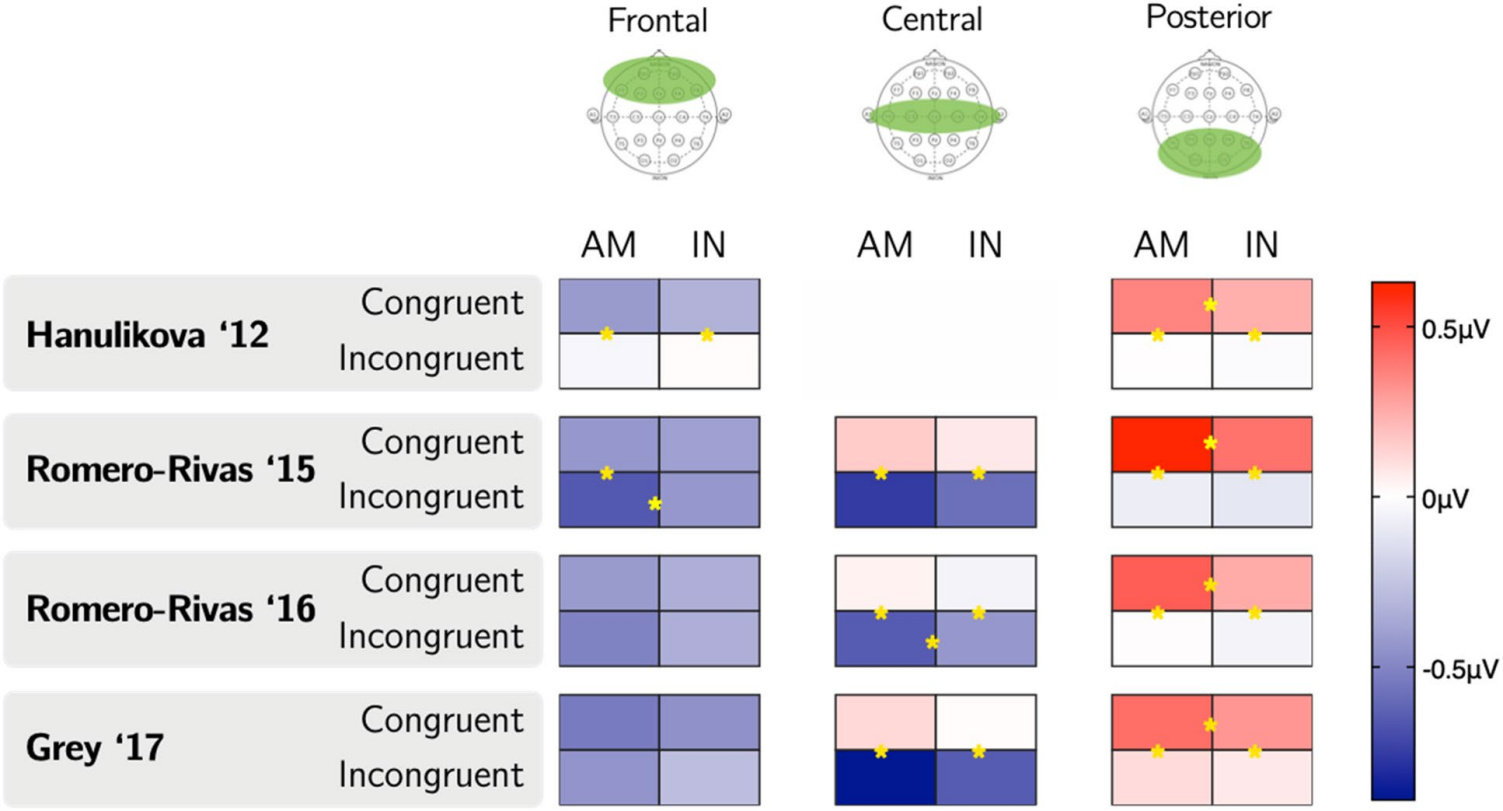
A graphical representation of average amplitudes in microvolts for each condition in the N400 time range for each parameter set, with a star on a line indicating a difference between conditions sharing that line significant at *p* < 0.05.

There was no three-way interaction between Accent, Congruence, and Region significant at *p* < 0.05 for any of the parameter sets. This was consistent with the G17 study but not the RR15 study, which found a significant interaction. To serve as a point of comparison to the RR15 study, we examined relevant comparisons in each region. A graphical representation of these comparisons is shown in Fig. 3. The greatest variation across parameter sets appears to exist frontally, which is incidentally where some studies have observed differential effects for in-group as compared to out-group speech. H12 and RR15 observed greater negativity to incongruent than congruent sentences in out-group but not in-group speech in the frontal region. By contrast, despite wide frontal variation in results across sets as seen in Fig. 3, we in no case observe a significantly greater frontal negativity to incongruence relative to congruence for out-group speech. Of note, across all parameter sets, responses to congruence in the posterior region were significantly more positive for the American accent than for the Indian accent. This was not explicitly noted in any of the studies, although visual inspection of signal in posterior electrodes presented in each study seems consistent in direction, if not in significance, with this result.

### A priori late (“P600”) range

**Figure 4:**
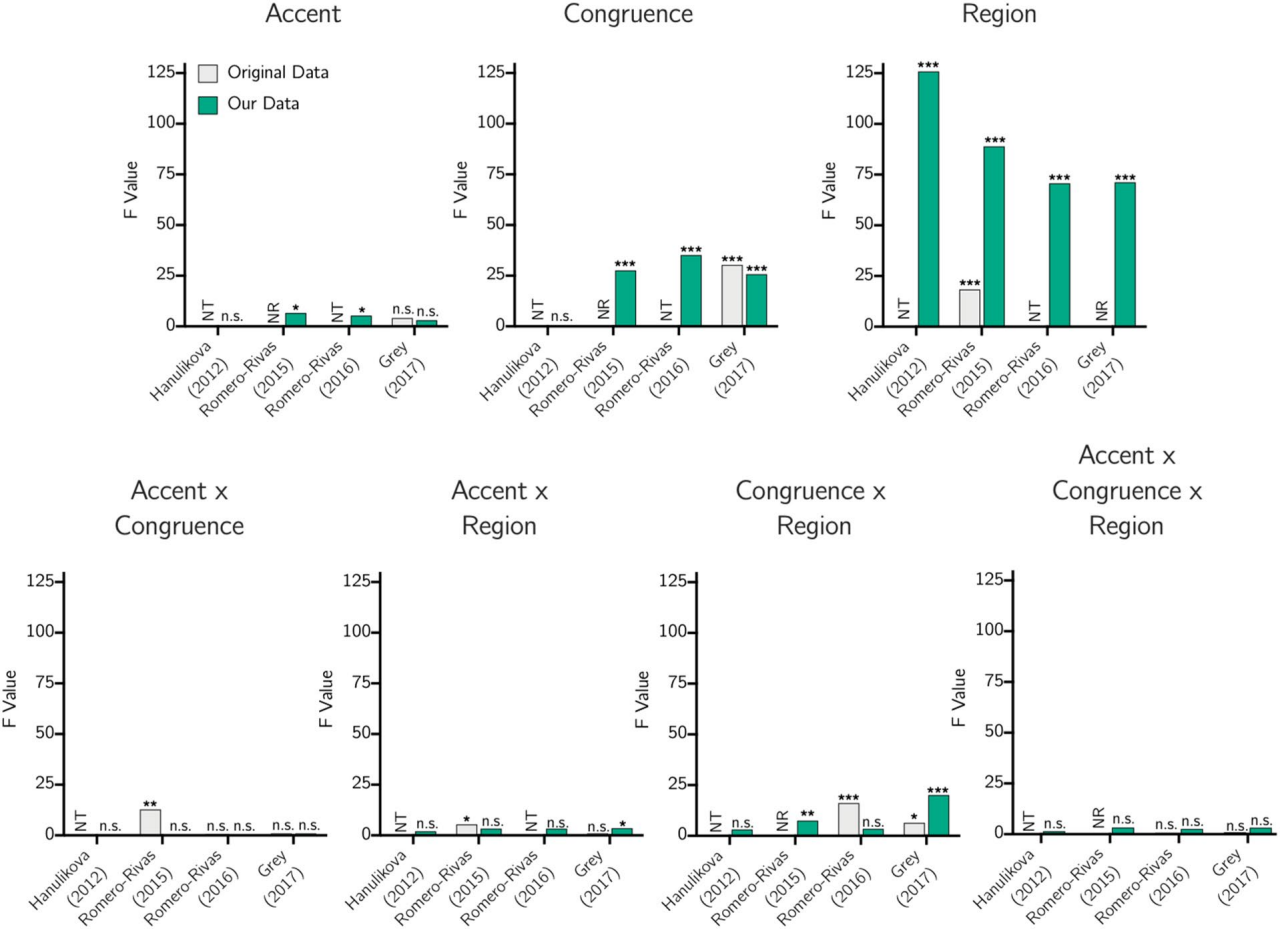
A graphical representation of F values for each main effect and interaction in the P600 time range found using each parameter set, either as reported in the original study (“original data”) or using our data (“our data”). Note: Stars indicate significance (*p < 0.05, **p < 0.01, ***p < 0.001), “n.s.” indicates not significant at *p* < 0.05, “NR” indicates the main effect or interaction was tested in the original study but a significant result or F value was not reported, and “NT” indicates testing of the main effect or interaction was not conducted in the original study in a way comparable with our approach.

We show F values for the ANOVA in the P600 time window presented in each of the studies when available, as well as the F values calculated using each study’s parameters with our data set, in Fig. 4.

We found a significant effect of Accent in the P600 range using the parameters of RR15 and RR16, an effect trending toward significance using the parameters of G17, and no effect using the parameters of H12. Neither RR15 nor RR16 reported an effect of Accent, but G17 did find an effect trending toward significance, though the direction of the effect is not made clear. Follow-up analysis revealed that responses were more negative to out-group speech than to in-group speech, with this difference significant at *p* < 0.01 for the RR15 set and *p* < 0.04 for the RR16 set, and trending toward significance (*p* < 0.10) for the G17 set.

We found a significant effect of Congruence using the parameters of RR15, RR16, and G17, but no effect using the parameters of H12. Follow-up analysis revealed responses were significantly (*p* < 0.001) more negative to incongruent sentences than to congruent sentences using the parameters of RR15, RR16, and G17. A significant effect of Congruence is consistent with G17, who found incongruence to elicit more negative responses than congruence, but not RR15.

We did not find a significant interaction of Accent and Congruence using any of the parameter sets. Follow-up analyses of our own data show this is because we see significantly (*p* < 0.001) more negative responses to incongruence relative to congruence for both accents in this time window for each parameter set except for H12, for which there is no difference between congruent and incongruent sentences in either accent. A more negative response to incongruence than congruence in both accents is consistent with G17. However, it is inconsistent with RR15, who found a significant interaction and, in follow-up analyses, a more positive response for incongruence than congruence in in-group speech but no difference in out-group speech.

We found an interaction between Accent and Region significant at *p* < 0.05 using the G17 parameters, as well as an interaction trending toward significance (*p* < 0.06) using the RR15 and RR16 parameters. G17 did not find an interaction between Accent and Region, but RR15 did, demonstrating in post hoc analysis that responses in this time range were more positive to in-group speech than out-group speech in the posterior region but not the frontal and central regions. Follow-up analysis of our data set revealed a similar phenomenon, with responses to in-group speech significantly more positive at *p* < 0.05 in the posterior region, and either significantly or trending toward significantly (*p* < 0.07) more positive in the central region, than to out-group speech for RR15, RR16, and G17.

**Figure 5:**
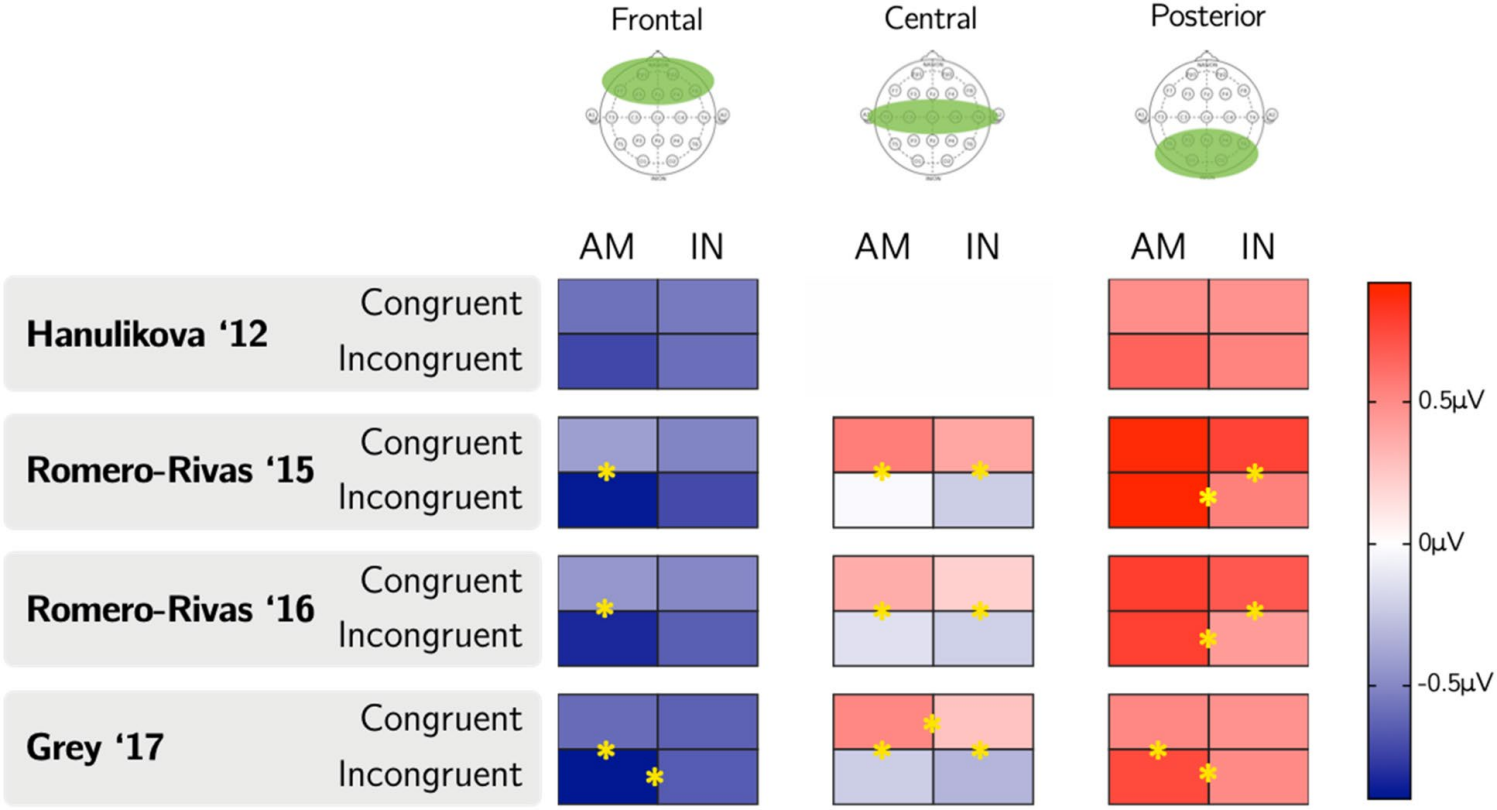
A graphical representation of average amplitudes in microvolts for each condition in the P600 time range for each parameter set, with a star on a line indicating a difference between conditions sharing that line significant at *p* < 0.05.

There was an interaction between Accent, Congruence, and Region significant at *p* = 0.05 for the G17 parameter set and trending toward significance for the analytical parameters of RR15 (*p* < 0.06) and RR16 (*p* < 0.11). RR15, RR16, and G17 did not find a significant three-way interaction in their respective studies. A graphical representation of individual comparisons is shown in Fig. 5. The most variation appears to exist in the posterior region, which has been the site of conflicting reports of a late positivity to incongruence^9,10,12^. Of note, for all parameter sets using three regions (RR15, RR16, and G17), posterior responses were more positive to incongruence in the American accent than in the Indian accent. This is consistent with the apparent late positivity in in-group but not out-group speech pointed out in the H12 results by RR15. Based on visual inspection of the results shown in posterior electrodes of RR15, RR16, G17, and H18, it is also consistent at least in direction, if not in significance, with these studies.

### Cluster-based permutation test

Non-parametric analysis was conducted by cluster-based permutation testing (CBPT), a highly sensitive approach used in H18 that avoids multiple comparisons and bias from a priori setting of analysis windows. It was conducted here separately from the previously reported analyses that used specific electrode clusters and time windows selected a priori. A separate test for each accent revealed that there was a significant effect to semantic congruence vs semantic incongruence in both in-group and out-group speech (*p* = 0.002). For in-group speech, this effect corresponded to a positive cluster approximately in the 180–700 ms time range in midline-neighboring electrodes in anterior, central, and posterior regions, although precise boundaries cannot be established by CBPT. This finding is broadly consistent with H18, who found a positive cluster between 200 and 900 ms for congruent as compared to incongruent sentences in in-group speech. For out-group speech, the cluster was approximately in the 310–740 ms time range in midline-neighboring electrodes in anterior, central, and posterior regions. This is temporally much broader than the cluster found by H18 for out-group speech, which was between 560 and 660 ms.

To investigate the interaction of congruence and accent effects, a difference wave between semantically congruent and incongruent sentences was computed separately for in-group and out-group speech. A third CBPT comparing the two difference waves found no significant clusters; this is consistent with H18, who did not find a significant cluster when comparing the difference waves for their two accents.

### Intelligibility, accentedness, and acceptability

As described in greater detail in the Methods section, a separate set of 32 participants completed a series of tasks designed to gauge the intelligibility, accentedness, and acceptability of the sentences used in the EEG study. There was no significant difference in intelligibility of the final (critical) word of the sentence for congruent sentences between the two accents (American: mean = 0.99, SD = 0.02; Indian: mean = 0.98, SD = 0.03; *p* = 0.29). This suggests that participants generally perceived the two accents as equally (and nearly completely) intelligible in the absence of semantic violations. Interestingly, there was a significant difference between accents in intelligibility of the final word for incongruent sentences, with higher accuracy for the American accent than the Indian accent (American: mean = 0.86, SD = 0.09; Indian: mean = 0.75, SD = 0.11; *p* < 0.001). This is consistent with previous work suggesting that listeners’ perceptions are more likely to be driven by context in out-group as compared to in-group speech^18,19^. While the difference is fairly small, it may have implications for differences in neural responses to incongruence in in-group and out-group speech. When asked to rate speakers on a scale on which 1 was “completely foreign” and 7 was “completely American,” participants rated the American accent as significantly more American than the Indian accent (American: mean = 6.77, SD = 0.28; Indian: mean = 2.39, SD = 0.57; *p* < 0.001). When asked to rate sentences on a scale on which 1 was “completely unacceptable” and 7 was “completely acceptable,” participants rated congruent sentences as significantly more acceptable than incongruent sentences (congruent: mean = 6.82, SD = 0.14; incongruent: mean = 2.24, SD = 0.62; *p* < 0.001). There was a small but significant difference in rated acceptability between accents for congruent sentences, with congruent sentences rated slightly more acceptable in the American than the Indian accent (American: mean = 6.90, SD = 0.10; Indian: mean = 6.73, SD = 0.23; *p* < 0.001), but there was no difference in rated acceptability between accents for incongruent sentences (American: mean = 2.23, SD = 0.67; Indian: mean = 2.23, SD = 0.63; *p* = 0.92).

## Discussion

Previous studies examining ERP responses to semantic congruence and incongruence in in-group and out-group accents have reported inconsistent results. In this study, we hoped to explore whether discrepancies might be driven by differences across studies in study design parameters, analytical parameters, or both by examining our own data set using the analytical parameters of each study. The same results across analytical parameter sets would suggest that only study design parameters drive differences between our study and other studies; a full match between each study’s results and our results derived using that study’s parameter set would suggest that only analytical parameters drive differences; varied outcomes across analytical parameters that do not fully match the studies from which those analytical parameters are derived would suggest both study design and analytical parameters drive differences.

The starkest effects we have observed, such as a posterior and central negativity in incongruent as compared to congruent conditions in the N400 window in both accents, persist across analytical methods. Some of these effects, such as a posterior negativity to incongruence in the in-group accent, have also been observed consistently across the studies of focus here and are generally consistent with classical N400 topography^15^. However, other findings that persist across the a priori analytical methods that we used on our own data set contrast with some previous results. In one example, in the N400 window, we found across all parameter sets with three regions that incongruent sentences spoken in an in-group accent elicited a greater negativity than incongruent sentences in an out-group accent; this agrees with the finding of Grey and van Hell^12^ but not Romero-Rivas et al.^10^, who found the opposite effect. In a second example, we found that incongruent sentences elicited greater negativity than congruent sentences in the N400 window for both accents across all parameter sets with three regions; this agrees with Romero-Rivas et al.^10^ but not Grey and van Hell^12^, who found that incongruent sentences elicited greater negativity than congruent sentences in in-group speech in this window. In a third example, we found a late negativity in the P600 window across parameter sets to incongruent as compared to congruent sentences in both accents across all parameter sets with three regions. This is consistent with Grey and van Hell^12^ but not Romero-Rivas et al.^10^, who found a late positivity to semantic violations in in-group speech but not out-group speech. Given that they persist across analytical parameter sets, these findings can be considered robust to our data set.

A couple of findings from our data that persist across analytical parameters were not broadly reported in the studies of focus here but seem, based on visual inspection of individual electrode data, to agree at least in direction with those studies. In one example, we found a more positive posterior response in the N400 window to congruent sentences in the American accent than the Indian accent across all parameter sets. In a second example, we observe that incongruent sentences evoke a more positive posterior response in the P600 range in the American accent as compared to the Indian accent in all parameter sets that use three regions. Though this latter finding is consistent with the data of Hanulíková et al.^9^ as noted by Romero-Rivas et al.^10^, neither of these findings was explicitly reported in any of the studies of focus here. However, visual inspection of signal in posterior electrodes presented in each of the five studies seems consistent at least in direction, if not in significance, with each finding. Given the presence of these trends across studies, these particular effects could perhaps be the focus of planned comparisons in future work.

As discussed by Romero-Rivas et al.^10^ in reference to the findings of Hanulíková et al.^9^, it is plausible that the increased negativity in the N400 window to incongruent speech in the in-group as compared to out-group accent reflects a greater initial perception of semantic violation in response to in-group speech, while the greater positive posterior response in the P600 window reflects a greater engagement of reanalysis processes aimed at making sense of the perceived violation. The greater positive posterior responses to congruence for in-group vs out-group speech in the N400 window, perhaps suggesting stronger perception of semantic match in in-group speech, could also be consistent with this explanation. One potential cause of these differences could be listeners’ phonetically driven reduced understanding of the Indian-accented speech as compared to the American-accented speech, with listeners having greater trouble recognizing individual frame or target words and processing sentences’ overall semantic content in Indian-accented speech, which could ultimately cause less perception of violation and thus less need to reanalyze that violation. This could be consistent with lower intelligibility for the final word in incongruent sentences in the Indian accent as compared to the American accent. However, it is unclear whether this reduced intelligibility translates to reduced perception of violation in this case, and an argument that differences are driven by intelligibility would be inconsistent with the observation of no difference in acceptability ratings between accents for incongruent sentences. Another potential cause could be differences in how individuals listen to members of their own social group as compared to members of another social group. For example, less interest in or more tolerance of the speech of an out-group speaker could result in less perceived violation and less reanalysis of that violation in out-group speech. Along these lines, Holt et al.^13^ observed that listeners who were more familiar with the out-group accent showed a greater N400 effect (and one more similar to that for in-group speech) than less familiar listeners, suggesting this could be because familiar listeners treated out-group speech more like in-group speech without giving out-group speakers “special treatment.” Future work should attempt to examine these potential phonetic and social causes of differences in responses to in-group and out-group accents.

The observation that certain findings in our data set differ from the findings of studies whose analytical parameters were used to derive them suggests that study design plays a role in driving these differences. While as highlighted in the introduction there are a number of dimensions along which the studies of focus differ in design from ours, we will discuss a few key dimensions and how variation along them might drive some of the variation in results we observe.

As mentioned previously, one major point of variation among the studies of focus here is participant and speaker background. Hanulíková et al.^9^ had native Dutch-speaking participants listen in Dutch to speakers with a native Dutch accent and a Turkish accent; Romero-Rivas et al.^10^ had presumably multilingual participants largely from Catalonia, an inherently multilingual locale, for whom Spanish was their dominant language listen in Spanish to speakers with Spanish, French, Greek, Italian, and Japanese accents; Grey and van Hell^12^ had English-speaking American monolinguals with limited out-group accent experience listen to American-accented and Chinese-accented English; Holt et al.^13^ had English-speaking Australian monolinguals in a major metropolitan area with likely significant out-group accent experience listen to Australian-accented and Chinese-accented English. Given that our study was conducted on English-speaking monolinguals in the San Francisco Bay Area at a university with a sizable international population, our participant sample is likely most similar to that of Holt et al.^13^; notably, our results largely match theirs when using their analytical method. The noted differences in listener and speaker background across studies are relevant to either social or phonetic causes of differences in processing of in-group and out-group accents. It is plausible that bilinguals would be able to relate more closely to speakers of another accent, as they themselves would have more experience as out-group speakers, and that they would also be able to understand more in the face of phonetic variation given their more varied linguistic experience. This could, for example, explain why we see greater negativity to incongruence in in-group vs out-group speech in the N400 window in our data and the data of Grey et al.^12^, both of which used English-speaking monolingual listeners, but not in the results of Romero-Rivas et al.^10^, who presumably used multilingual listeners. Listener exposure to out-group accents is another variable relevant to either social or phonetic causes, as it could make listeners more likely to understand the phonetic properties of an out-group accent^19,20^, or more likely to relate closely to out-group speakers and thus judge their speech more like in-group speech. Exposure could explain why we see an N400 effect to incongruence versus congruence for out-group speech in our data, the study of Romero-Rivas et al.^10^, and the out-group-familiar listeners in Holt et al.^13^, but not in Grey and van Hell^12^ or the out-group-unfamiliar listeners of Holt et al.^13^ (though it should be noted that Grey and van Hell^12^ did observe a late negativity to incongruence vs congruence for out-group speech). For potential social and phonetic causes alike, the interaction between speaker group and listener group is also important to consider. The social roles of a Turkish accent in the Netherlands, a Japanese accent in Catalonia, and an Indian accent in California all likely differ. Additionally, previous work has suggested that individuals have greater intelligibility for accents that are more phonologically similar to their own^20^, so we might expect differences for an English speaker listening to a Chinese or Indian accent as compared to a Spanish speaker listening to a collection of accents that include Italian and Greek.

Second, accent aside, there is significant variation across the studies of focus here in other linguistic features of the experimental stimuli used. Hanulíková et al.^9^ and Grey and van Hell^12^ included both a semantic manipulation and a grammatical manipulation, while Romero-Rivas et al.^10^, Romero-Rivas et al.^11^, and Holt et al.^13^ included just a semantic manipulation. While most studies used a violation condition and a non-violation condition, Romero-Rivas et al.^11^ used varying amounts of semantic appropriateness but did not include a semantic violation condition. Critical word position also varied, with Hanulíková et al.^9^ placing the target word at least five syllables before the end of the sentence; Romero-Rivas et al.^10^, Romero-Rivas et al.^10^, and Grey and van Hell^12^ using varied placing across sentences; and Holt et al.^13^ placing the target word at the end of the sentence. Given that context plays a differential role in perception of in-group and out-group speech^18,19^, the extent to which context is built up when the critical word is introduced (and when the clock usually starts for the placement of the N400 window) could drive differences across studies. Our design most closely matched that of Holt et al.^13^ on these features, as we included only a semantic manipulation and put critical words at the end of sentences, and it is again notable that our results broadly paralleled theirs.

Effects between other conditions at particular region/time range pairings depend on the analytical method used. Observations for these particular comparisons in some cases differentiate studies in this literature from each other and are highlighted in some studies as distinguishing processing of in-group and out-group speech. In one example, we found more negative amplitudes to congruent sentences for the out-group accent than the in-group accent when using the Romero-Rivas et al.^10^ parameters, which agrees with the result from the first block of their study, and which they cite as evidence of listener adaptation to out-group speech over time. However, we did not find this for parameters from any of the other studies, which themselves do not report a difference. In another example, Hanulíková et al.^9^ and Romero-Rivas et al.^10^ identified a frontal negativity to semantic incongruence in the N400 window for out-group speech that was not present for in-group speech. While we did not find a frontal negativity to incongruence for the out-group speech in the N400 range using any of the a priori parameter sets on our data, we did find that whether a significant difference was observed frontally between congruent and incongruent speech depended on parameter set. Both Hanulíková et al.^9^ and Romero-Rivas et al.^10^ highlight this frontal difference as a feature distinguishing processing of in-group and out-group speech, but it appears that at least in our data set the observation of this difference is dependent on the analytical parameters used.

Given that by using each study’s individual analytical parameters we were able to reproduce certain findings but not others that differ across studies, it thus appears likely that some but certainly not all of the differences in results observed across studies may be attributable to differences in analytical parameters, with other differences driven by variation in study design and execution. Of course, because we only used data from our own study in our analysis, we can only conclusively assert the relative role of these two factors when comparing our study’s results to those of the other studies; future work would be helpful in conclusively establishing the role of these factors more broadly in the literature. It is also difficult to determine from our results alone the relative weighting of these two factors. Since a decent amount of variation is driven by analytical parameters, coalescing around common analytical techniques could give this literature a shared reference point that will help in the identification of real effects. This could be achieved through following the suggestion of Luck and Gaspelin^21^ to establish a priori windows before looking at data (and preferably, for the sake of a common language, that are consistent across studies), or to use mass univariate tests like cluster-based permutation tests to find effects in an unbiased fashion without a huge number of comparisons. Interestingly, the results in our data set derived from the cluster-based permutation test similar to that used by Holt et al.^13^ are largely consistent with their results. At the very least, given how much difference analytical parameters can drive in this area, it would be worthwhile for researchers to provide clear justification of their choice of analytical parameters and to take analytical parameters into account when drawing comparisons between studies. Additionally, since study design parameters also seem to play a large role in variation in results, future studies could attempt to contrast variables most likely to cause differences, such as the particular out-group accent used, the language background of participants, or the amount of participants’ exposure to the accents tested.

These are still early days for neural studies of accented speech, and much remains to be determined about what effects actually exist and the degree to which those effects are being influenced by factors like properties of the speaker and listener, properties of the stimulus materials, and features of the analysis process. It is as yet unclear whether there are universal differences in processing in-group and out-group speech that transcend the features of specific situations, but we believe this study can be a step in the direction of determining that. We hope that this study can help researchers working in this field as they are considering how to analyze their data. We also hope it prompts discussion over how differences in evoked potentials between in-group and out-group speech can best be investigated given what still remains to be determined about their time course and topography.

## Methods

### Participants

Forty-nine participants took part in the EEG experiment. Participants received $75 for taking part in the study. Participants were native speakers of English who had been born and raised in the US or Canada (48 from the US, one from Canada), did not have native-like fluency in a language other than English, and had not spent more than one year outside North America. None of them reported any hearing or neurological abnormalities. All participants provided informed consent to participate in this study, which was approved by and carried out in accordance with the guidelines and regulations of the Institutional Review Board of Stanford University.

A separate set of 32 participants adhering to the criteria described above were recruited to participate in a brief task intended to assess the intelligibility and accentedness of the sentences used in the EEG study.

### Stimuli

Experimental stimuli of focus in this study comprised a total of 216 sentences. Half of these sentences were spoken in English by a native speaker of Standard American English, and the other half were spoken in English by a native Hindi speaker born and raised in India. In an attempt to increase generalizability, two speakers of each accent were used in the experiment, but each participant only heard one native American English speaker and one native Hindi speaker (with a total of four different combinations distributed equally across participants). There was no substantive or systematic difference in accent or region of origin between the speakers who generated the American English stimuli and the participants in the EEG experiment.

Half of the sentences spoken by each speaker were semantically congruent, and the other half were semantically incongruent. For semantically incongruent sentences, the violation always occurred in the final word. This gave a total of 54 sentences for each condition (accent × congruence). Congruent sentences were drawn from Block and Baldwin^22^, with an average cloze probability of 0.84 and minimum cloze probability of 0.6. Incongruent sentences frames were also drawn from Block and Baldwin^22^; final words from the sentences in this set were removed, their order was randomized, and they were then added onto the original sentence frames. Sentences were randomly sorted into congruent and incongruent sets, and the set of 54 congruent and 54 incongruent sentences spoken by each speaker was counterbalanced across participants. During the same paradigm, interleaved with the sentences of focus in this study, participants heard an additional 432 sentences ending with words whose initial consonants were either /p/, /b/, /k/, or /g/; the analysis of responses to these stimuli will be the focus of another paper.

Sentences were recorded and edited with Audacity at 44.1 kHz, in 64 bits, and in monaural sound. Sentence order was randomized, with speakers reading alternating sets of congruent and incongruent sentences to avoid systematic differences in their recording. Sentences were balanced on duration between accents (American: mean = 2.63 s, SD = 0.48; Indian: mean = 2.65 s, SD = 0.43) and between congruence types (congruent: mean = 2.65 s, SD = 0.45; incongruent: mean = 2.63 s, SD = 0.46).

### EEG recording

Prior to the recording, participants listened to two separate passages of between 4 and 5 min voiced by each of the two speakers, and after each passage they were asked to complete a writing task that took approximately 5 min. In this exercise, which will be the focus of another paper, participants were randomly assigned to two separate groups differentiated by the extent to which they were encouraged to take the out-group speaker’s perspective by the listening passages and writing task. However, for the sake of the current paper’s analysis intended to examine the role of analytical parameters and identify results characteristic of the entire participant sample, the data from all participants were collapsed together.

Before the EEG recording began, participants were told the two speakers they had just heard from in the listening passages would be saying sentences about their world, their lives, and their friends. Participants were instructed to listen carefully and attentively to the sentences. In addition, to ensure they remained engaged, participants were asked to press a button on a button box when they heard a sentence identical to the sentence two previous to it. Half of participants in each condition were asked to press the left button on a button box using their left hand, and the other half of participants were asked to press the right button on the button box using their right hand.

During the recording, participants sat in a comfortable chair in an electrically and acoustically isolated room. They watched a black screen at a distance of approximately 1.5 m while listening to sentences presented in series. During the recording, participants heard the 216 sentences of focus in this study, as well as an additional 432 sentences designed to interrogate particular phonetic features that will be the focus of another paper. The order of these 648 sentences was randomized and broken into eight blocks of 81 sentences each. In each block, between 6 and 10 sentence repeats were added; each repeat was added two sentences after the original sentence, with a total of 10% of all original sentences having repeats across the blocks. As a result, a total of 712 sentence items were used in the entire recording (648 original sentences + 64 repeated sentences). At the beginning of each trial, a white cross appeared in the center of the screen, and the sentence began playing a randomly jittered amount of time between 600 and 800 ms later, averaging out to 700 ms across trials. The cross remained on the screen while the sentence played and for a randomly jittered amount of time between 1000 and 1200 ms after the sentence ended, averaging out to 1100 ms across trials. The cross then disappeared and the screen was entirely black for 1000 ms, at which point the next trial began with the appearance of a cross. Each of the eight blocks was between 8 and 9 min in length, and the total recording time was approximately 70 min. The experimenter checked on participants after each block and gave them the opportunity to take a break at any time between blocks. They then completed a series of behavioral tasks that will be the focus of another paper.

The EEG signal was recorded using the Neuroscan SymAmpRT amplifier and Curry 7 acquisition software with a whole-head 64-channel Quikcap containing electrodes in standard 10–20 locations. The sampling rate was 500 Hz. Scalp electrodes were referenced to an electrode located on the midline between CPz and Cz during the recording. Electrodes above and below the left eye were used to detect vertical EOG, and electrodes beside each eye were used to detect horizontal EOG. Care was taken to keep impedances at each electrode below 10 kΩ.

### Intelligibility, accentedness, and acceptability task

A separate set of 32 participants listened to sentences used in the EEG recording and for each sentence provided responses intended to gauge intelligibility and accentedness of the speech and acceptability of the semantic content of the sentence. Each participant heard a random set of 108 sentences total from across the four speakers, with each speaker-sentence combination used twice across all participants. After each sentence, the participant was first asked to type out the final word of the sentence, a common task used to gauge intelligibility. Next, the participant was asked to indicate on a scale between 1 and 7 how foreign or American the speaker sounded, with 1 being “completely foreign” and 7 being “completely American.” Finally, the participant was asked to indicate how acceptable the sentence was or how much sense it made, with 1 being “completely unacceptable” and 7 being “completely acceptable”.

### EEG and ERP analyses

The EEG signal was filtered offline between 0.1 and 50 Hz. Vertical and horizontal EOG were used to detect eye artifacts (blinks and eye movement) for each participant and to remove these artifacts from the continuous EEG signal in each of the eight blocks using source space projection in the Brainstorm toolbox^23^.

The EEG signal was broken into epochs starting 200 ms before and ending 1200 ms after the onset of the sentence’s final word. For each epoch, EEG waveforms were baseline corrected to a 200 ms period before the onset of the sentence’s final word. For epoched single trial data, channels exceeding − 100 μV and 100 μV were excluded from the analysis of the epoch. Epochs were then averaged by condition for each participant. Mean amplitudes for each condition in each participant were then computed for each analysis parameter set mentioned in Table 1. Repeated measures ANOVAs were then run separately for each time window in each analysis parameter set using the following factors: accent (American or Indian), semantic status (congruent or incongruent), and region (frontal or posterior OR frontal, central, or posterior, depending on the analysis parameter set). Greenhouse–Geisser correction was used to correct for sphericity.

Cluster-Based Permutation Tests (CBPTs) were performed for each factor of interest over the entire time window of the EEG signal (− 200 ms to 1200 ms) as described in Maris & Oostenveld^24^ and implemented in Brainstorm. To conduct a CBPT for a given factor, grand-averaged data across all relevant trials were generated for each subject at each level of the factor. Then, a *t*-statistic was generated for the difference in EEG signal at all time points and all electrodes. Points with *t* values between 2 and − 2 were discarded. The remaining points were clustered in a two-dimensional (time × electrode) space, with a minimum of 2 neighbors required for cluster growth. The resulting clusters were each assigned a “mass” statistic equal to the sum of the *t*-values of their member points. Finally, the CBPT was considered significant if the largest cluster mass thus obtained was greater than 95% of all absolute cluster masses obtained after 1000 iterations of a Monte-Carlo-style randomization process.

The datasets generated and analysed during the current study are available from the corresponding author on reasonable request.

## Supplementary Information

Supplementary Information.
