# Supplementary material for: Replicability of neural responses to speech accent is driven by study design and analytical parameters
Source: Sci Rep. 2021 Feb 26;11:4777. doi: 10.1038/s41598-021-82782-4 (PMC7910471; doi:10.1038/s41598-021-82782-4)
Supplement: Supplementary file 1 — Supplementary Information. [file 41598_2021_82782_MOESM1_ESM.docx]

**SUPPLEMENTARY MATERIAL**

**Title**

Disagreement in measured neural responses to speech accent is driven by study design and analytical parameters

**Author list and affiliations**

C. Benjamin Strauber* (Stanford University), Lestat R. Ali (Harvard Medical School), Takako Fujioka (Stanford University), Candace Thille (Stanford University), Bruce D. McCandliss (Stanford University)

*Correspondence to strauber@stanford.edu

Supplementary Table 1 -- ANOVA results reported in two studies (“their data set”) and derived using each study’s analytical parameters on our data (“our data set”) for the N400 time range

|  | Hanulíková 2012 | | | Romero-Rivas 2015 | | | Romero-Rivas 2016 | | | Grey 2017 | | |
| --- | --- | --- | --- | --- | --- | --- | --- | --- | --- | --- | --- | --- |
|  | df | F | p | df | F | p | df | F | p | df | F | p |
| Accent |  |  |  |  |  |  |  |  |  |  |  |  |
| Our data set | 1,48 | 0.04 | 0.84 | 1,48 | 0.15 | 0.70 | 1,48 | 0.48 | 0.49 | 1,48 | 1.29 | 0.26 |
| Their data set | NT | NT | NT | NR | NR | NR | NT | NT | NT | 1,28 | <1 | NR |
| Congruence |  |  |  |  |  |  |  |  |  |  |  |  |
| Our data set | 1,48 | 2.04 | 0.16 | 1,48 | 96.90 | <0.001 | 1,48 | 67.66 | <0.001 | 1,48 | 65.16 | <0.001 |
| Their data set | NT | NT | NT | 1,19 | 4.25 | <0.05 | NT | NT | NT | 1,28 | 63.41 | <0.001 |
| Region |  |  |  |  |  |  |  |  |  |  |  |  |
| Our data set | 1,48 | 29.7 | <0.001 | 2,96 | 55.33 | <0.001 | 2,96 | 45.21 | <0.001 | 2,96 | 45.02 | <0.001 |
| Their data set | NT | NT | NT | NR | NR | NR | NT | NT | NT | NR | NR | NR |
| Accent x Congruence |  |  |  |  |  |  |  |  |  |  |  |  |
| Our data set | 1,48 | 0.97 | 0.33 | 1,48 | 5.67 | 0.02 | 1,48 | 8.34 | 0.006 | 1,48 | 5.31 | 0.03 |
| Their data set | NT | NT | NT | 1,19 | 5.41 | <0.05 | 1,32 | 2.53 | 0.12 | 1,28 | 10.12 | <0.05 |
| Accent x Region |  |  |  |  |  |  |  |  |  |  |  |  |
| Our data set | 1,48 | 2.44 | 0.13 | 2,96 | 4.61 | 0.02 | 2,96 | 4.13 | 0.03 | 2,96 | 2.21 | 0.12 |
| Their data set | NT | NT | NT | NR | NR | NR | NT | NT | NT | 2,56 | <1 | NR |
| Congruence x Region |  |  |  |  |  |  |  |  |  |  |  |  |
| Our data set | 1,48 | 75.1 | <0.001 | 2,96 | 22.55 | <0.001 | 2,96 | 13.09 | <0.001 | 2,96 | 39.05 | <0.001 |
| Their data set | NT | NT | NT | 2,38 | 7.11 | <0.05 | 2,64 | 31.4 | <0.001 | 2,56 | 5.17 | NR |
| Accent x Congruence x Region |  |  |  |  |  |  |  |  |  |  |  |  |
| Our data set | 1,48 | 1.90 | 0.17 | 2,96 | 0.16 | 0.81 | 2,96 | 1.01 | 0.35 | 2,96 | 1.56 | 0.22 |
| Their data set | NT | NT | NT | 2,38 | 3.69 | <0.05 | 2,64 | 0.38 | 0.55 | 2,56 | <1 | NR |

Supplementary Table 2 -- ANOVA results reported in two studies (“their data set”) and derived using each study’s analytical parameters on our data (“our data set”) for the P600 time range

|  | Hanulíková 2012 | | | Romero-Rivas 2015 | | | Romero-Rivas 2016 | | | Grey 2017 | | |
| --- | --- | --- | --- | --- | --- | --- | --- | --- | --- | --- | --- | --- |
|  | df | F | p | df | F | p | df | F | p | df | F | p |
| Accent |  |  |  |  |  |  |  |  |  |  |  |  |
| Our data set | 1,48 | 0.06 | 0.81 | 1,48 | 6.62 | 0.01 | 1,48 | 5.35 | 0.03 | 1,48 | 3.06 | 0.09 |
| Their data set | NT | NT | NT | NR | NR | NR | NT | NT | NT | 1,28 | 4.12 | <0.10 |
| Congruence |  |  |  |  |  |  |  |  |  |  |  |  |
| Our data set | 1,48 | 0.10 | 0.76 | 1,48 | 27.59 | <0.001 | 1,48 | 35.21 | <0.001 | 1,48 | 25.72 | <0.001 |
| Their data set | NT | NT | NT | NR | NR | NR | NT | NT | NT | 1,28 | 30.30 | <0.001 |
| Region |  |  |  |  |  |  |  |  |  |  |  |  |
| Our data set | 1,48 | 125.9 | <0.001 | 2,96 | 88.9 | <0.001 | 2,96 | 70.72 | <0.001 | 2,96 | 71.25 | <0.001 |
| Their data set | NT | NT | NT | 2,38 | 18.39 | <0.001 | NT | NT | NT | NR | NR | NR |
| Accent x Congruence |  |  |  |  |  |  |  |  |  |  |  |  |
| Our data set | 1,48 | 0.01 | 0.91 | 1,48 | 0.01 | 0.93 | 1,48 | 0.10 | 0.75 | 1,48 | 1.00 | 0.33 |
| Their data set | NT | NT | NT | 1,19 | 12.78 | <0.01 | 1,32 | 0.71 | 0.41 | 1,28 | <1 | NR |
| Accent x Region |  |  |  |  |  |  |  |  |  |  |  |  |
| Our data set | 1,48 | 2.06 | 0.16 | 2,96 | 3.34 | 0.05 | 2,96 | 3.34 | 0.06 | 2,96 | 3.57 | 0.04 |
| Their data set | NT | NT | NT | 2,38 | 5.38 | <0.05 | NT | NT | NT | 1,28 | <1 | NR |
| Congruence x Region |  |  |  |  |  |  |  |  |  |  |  |  |
| Our data set | 1,48 | 3.10 | 0.08 | 2,96 | 7.50 | 0.003 | 2,96 | 3.46 | 0.05 | 2,96 | 20.07 | <0.001 |
| Their data set | NT | NT | NT | NR | NR | NR | 2,64 | 16.13 | <0.001 | 2,56 | 6.44 | <0.05 |
| Accent x Congruence x Region |  |  |  |  |  |  |  |  |  |  |  |  |
| Our data set | 1,48 | 1.45 | 0.24 | 2,96 | 3.28 | 0.06 | 2,96 | 2.60 | 0.10 | 2,96 | 3.17 | 0.05 |
| Their data set | NT | NT | NT | NR | NR | NR | 2,64 | 0.66 | 0.44 | 2,56 | <1 | NR |

Supplementary Table 3 -- Averages (microvolts) in N400 range for our data set using different papers’ parameters

|  | Frontal | | Central | | Posterior | |
| --- | --- | --- | --- | --- | --- | --- |
|  | AM | IN | AM | IN | AM | IN |
| Hanulíková (2012) |  |  |  |  |  |  |
| Congruent | -0.42 | -0.33 |  |  | 0.36 | 0.24 |
| Incongruent | -0.04 | 0.01 |  |  | 0.001 | -0.02 |
| Romero-Rivas (2015) |  |  |  |  |  |  |
| Congruent | -0.43 | -0.40 | 0.16 | 0.07 | 0.63 | 0.41 |
| Incongruent | -0.66 | -0.43 | -0.76 | -0.58 | -0.08 | -0.11 |
| Romero-Rivas (2016) |  |  |  |  |  |  |
| Congruent | -0.42 | -0.34 | 0.04 | -0.05 | 0.46 | 0.25 |
| Incongruent | -0.51 | -0.34 | -0.65 | -0.43 | 0.003 | -0.05 |
| Grey (2017) |  |  |  |  |  |  |
| Congruent | -0.54 | -0.46 | 0.12 | 0.01 | 0.42 | 0.31 |
| Incongruent | -0.45 | -0.28 | -0.89 | -0.65 | 0.11 | 0.07 |

Supplementary Table 4 -- Averages (microvolts) in P600 range for our data set using different papers’ parameters

|  | Frontal | | Central | | Posterior | |
| --- | --- | --- | --- | --- | --- | --- |
| Hanulíková (2012) | AM | IN | AM | IN | AM | IN |
| Congruent | -0.58 | -0.55 |  |  | 0.49 | 0.47 |
| Incongruent | -0.73 | -0.59 |  |  | 0.65 | 0.53 |
| Romero-Rivas (2015) |  |  |  |  |  |  |
| Congruent | -0.41 | -0.51 | 0.55 | 0.39 | 0.88 | 0.77 |
| Incongruent | -0.89 | -0.72 | -0.03 | -0.22 | 0.92 | 0.54 |
| Romero-Rivas (2016) |  |  |  |  |  |  |
| Congruent | -0.44 | -0.50 | 0.36 | 0.21 | 0.79 | 0.69 |
| Incongruent | -0.83 | -0.65 | -0.14 | -0.21 | 0.78 | 0.43 |
| Grey (2017) |  |  |  |  |  |  |
| Congruent | -0.60 | -0.64 | 0.51 | 0.27 | 0.51 | 0.47 |
| Incongruent | -0.90 | -0.66 | -0.22 | -0.32 | 0.74 | 0.50 |
